# Effectiveness and cost-effectiveness of a loyalty scheme for physical activity behaviour change maintenance: results from a cluster randomised controlled trial

**DOI:** 10.1186/s12966-018-0758-1

**Published:** 2018-12-12

**Authors:** Ruth F. Hunter, Jennifer M. Murray, Aisling Gough, Jianjun Tang, Christopher C. Patterson, David P. French, Emma McIntosh, Yiqiao Xin, Frank Kee, on behalf of the Physical Activity Loyalty (PAL) Study team

**Affiliations:** 1 UKCRC Centre of Excellence for Public Health Research (NI)/Centre for Public Health, Queen’s University Belfast, Grosvenor Road, Belfast, BT12 6BJ Northern Ireland; 2 School of Agricultural Economics and Rural Development, Renmin University of China, Beijing, China; 3 School of Health Sciences, University of Manchester, Manchester, England; 4 Health Economics and Health Technology Assessment, University of Glasgow, Glasgow, Scotland

**Keywords:** Physical activity, Adults, Intervention, Behaviour change, Incentives, Effectiveness, Cost-effectiveness

## Abstract

**Background:**

We evaluated the effectiveness and cost-effectiveness of a loyalty scheme based intervention involving rewards for increasing physical activity in public sector employees.

**Methods:**

A cluster randomised wait-list controlled trial in public sector organisations in Northern Ireland. We randomly assigned clusters (1:1) using a computer generated random sequence. Researchers were masked to allocation, but participants were not. Employees aged 18–65 years with no self-reported medical contraindications to physical activity were included. The Physical Activity Loyalty Scheme (PAL) intervention was based on high-street loyalty cards where participants earned points for minutes of activity that could be redeemed for rewards, complemented by evidence-based behaviour change techniques. The primary outcome was objectively measured mean steps/day at 6 months using a validated pedometer (Yamax Digi-Walker CW-701) over 7 days, assessed with intention to treat analysis. Secondary outcomes included health, mental wellbeing, quality of life, work absenteeism and presenteeism, and use of healthcare resources. Cost-effectiveness, cost-benefit and mediation analyses were conducted. Trial registered with Current Controlled Trials, number ISRCTN17975376.

**Results:**

Between September 2014 and October 2015, we recruited and randomly assigned 37 clusters (from nine organisations; mean clusters per organisation = four) and 853 participants to the intervention (*n* = 19 with 457 participants) or control group (*n* = 18 with 396 participants). Primary outcome data were available for 249 (54·4%) intervention and 236 (59·6%) control participants. Mean steps/day were significantly lower in the intervention vs control group (adjusted mean difference = − 336, 95% CI: -612 to − 60, *p* = 0·02) at 6 months. Participants redeemed only 39% (SD 43%) of their earned points. Using the Quality Adjusted Life Year outcome, the intervention was not cost effective from an NHS/PSS perspective. A net cost analysis from an employer perspective demonstrated the intervention group was associated with a mean of 2·97 h less absenteeism over a 4 week period (p = 0·62), which could result in net savings ranging from £66 to £735 depending on the wage rate employed. At 4-weeks post-baseline there were significant increases in identified regulation, integrated regulation, intrinsic motivation, social norms and intentions in intervention compared to control participants.

**Conclusions:**

Our mixed results pose challenges that are too infrequently exposed in public heath intervention trials. Although the intervention successfully altered several hypothesised mediating constructs it did not translate into long-term behaviour change. Our incentive level may have been too low to incentivise change, despite being designed a priori by a Contingent Valuation Survey. There were also major re-structuring of several organisations which presented significant implementation challenges, and technical limitations.

**Trial registration:**

ISRCTN17975376 (Registered 19/09/2014).

**Electronic supplementary material:**

The online version of this article (10.1186/s12966-018-0758-1) contains supplementary material, which is available to authorized users.

## Introduction

There is a strong business case for investing in the health and wellbeing of the workforce [1]. It is estimated that for every £1 invested in workplace health and wellbeing, there is a potential return of over £4 as a consequence of reductions in absenteeism and improvements in productivity [2], with a possible dividend to the economy in the region of £30 billion annually [1]. With increasing numbers of inactive office-based occupations, improvements in physical activity (PA) levels may contribute positively to wellbeing, mental health, absenteeism and productivity. Evidence to support the effectiveness and cost-effectiveness of such interventions is mixed, with previous meta-analyses of workplace PA interventions showing small, positive, short-term effects on PA levels, but an absence of evidence on maintenance and cost-effectiveness from different perspectives [3–9].

The lack of evidence for the long-term effectiveness and cost-effectiveness of PA interventions is not unique to workplace interventions. A systematic review found that PA interventions targeting adults showed small positive effect sizes between 6 and 15 months follow-up (SMD 0·20–0·28), with few studies thereafter [10]. A further systematic review demonstrated that there were distinct mediators of PA behaviour change during initiation (first 6 months of a behaviour) and maintenance (beyond 6 months of a behaviour) phases [11]. This research points to the need to design interventions that purposefully support the transition from initiation to maintenance of behaviour change which involves distinct theoretical and implementation approaches.

Our earlier research demonstrated the potential effectiveness and cost-effectiveness of a workplace PA intervention in a pilot quasi-experiment, collaborating with the business sector, and purposefully developed on a sustainable ‘business’ model [12, 13]. In partnership with retailers, we adapted the general underlying business principles of a loyalty scheme, an existing mechanism that supports behaviour change in the private sector, and applied it to a public health setting. In the business sector, some loyalty schemes encourage repeated behaviour (i.e., loyalty), such as shopping at a particular retailer, by rewarding participants for their repeated business by collecting points and the opportunity to convert these into subsequent rewards, such as, retail vouchers. Using similar principles, we developed the “Physical Activity Loyalty Card” whereby participants earned points for minutes of PA, which could then be redeemed for rewards. Our pilot data provided evidence to support the potential of such an intervention to impact positively on workplace health and wellbeing, and provide economic benefits from a public health, employer and retailer perspective [12, 13].

Consequently, using pilot data [12, 13] we developed a pragmatic intervention primarily aimed at increasing PA levels among office-based public sector employees in Northern Ireland [14]. The intervention incorporated evidence-based behaviour change techniques, and was underpinned by relevant theory. A cluster RCT design was employed to minimise the risk of contamination of the intervention between the trial groups. Our aims were to compare the effectiveness and cost-effectiveness of the intervention versus the waiting-list control group. A further aim was to understand the underpinning mechanisms of PA behaviour change, through process analysis of hypothesised mediating variables.

## Methods

### Study design and participants

The study was a cluster RCT, with a parallel mediation analysis and economic evaluation.

Nine public sector organisations in two cities (Belfast and Lisburn) in Northern Ireland were purposively sampled from those within a 2 km radius of the city centre or which could offer PA opportunities within a 2 km radius of their location and had a minimum of 100 employees in predominantly office-based occupations. Meetings were held with senior management of these organisations to explain the study purpose and practicalities.

Participants were healthy adults working in office-based occupations in public sector organisations. Recruitment methods were email invitations to employees and posters placed around each workplace advertising the study [12]. Potential participants were able to access further information (including the Participant Information Sheet) and register their interest to participate on the study website. They were asked to complete a screening questionnaire via the study website or by telephone, to confirm their eligibility, based on the following inclusion criteria: based at recruited worksite at least 4 h/day (within core hours of 8 am-6 pm) on at least 3 days/week, current contract anticipated to last for the duration of the study, access to internet at work, able to give informed consent, and no self-reported recent medical events that would limit ability to participate in PA (assessed using the Physical Activity Readiness Questionnaire, PAR-Q).

The trial was delivered according to the updated published protocol [14]. Ethics approval was obtained from the Office for Research Ethics Committee Northern Ireland (ORECNI) (reference number 14/NI/0090; approval granted May 21, 2014). Local NHS Health and Social Care Trust approvals were obtained from Research Governance departments before the start of recruitment.

### Randomisation and masking

Clusters were randomly assigned to the intervention group or waiting-list control group (1:1). Clusters were the smallest work groups or units (e.g. a large open plan office) within each participating organisation. A random allocation sequence was drawn up by the trial statistician using a computer generated random sequence, and group allocation was stratified to ensure a similar number of clusters of each size and type (i.e. small organisations < 20 employees; medium organisations 20–50 employees; large organisations > 50 employees; school) in both intervention and control groups. Allocations were only accessible by an independent trial statistician and those delivering the intervention until after the trial had finished. The outcome of the randomisation was communicated to participants by email after baseline measurements were complete.

It was not possible to blind participants to group allocation. Research staff collecting the outcome data were masked to allocation, as were the statisticians and health economists conducting the analysis (the randomisation variable was unlabelled in the main dataset).

Eligible participants were introduced to the study by the research team and gave written informed consent prior to completion of baseline data collection and before group allocation was assigned.

### Procedures

Intervention components included the provision of points which could be converted into rewards (retail vouchers) contingent on meeting PA behaviour goals. The PAL Scheme integrated a PA remote tracking system with web-based monitoring and evidence-based behaviour change tools such as self-monitoring and goal-setting.

The 6 month intervention involved placing wifi beacons (sensors) at specific locations in the vicinity of participating workplaces to encourage PA within a 2 km radius of participants’ worksites (Additional file 1). The wifi beacons were placed at locations along footpaths, in local parks, leisure centres, shopping malls, bus stops and train stations. Maps of various walking routes and details about PA opportunities tailored to the workplace were provided on the study website. Participants’ PA was logged when they engaged in PA within an approximate 25 m radius of the wifi beacons carrying their PAL keyfob. The place, date and time of the bout of PA was logged. Participants could access their account on the study website and receive real-time feedback on the number of minutes of PA logged by the tracking system. Participants were encouraged to undertake 150 min/week of PA which was in line with current guidelines [15]. Minutes of PA were converted to points (1 point for 1 min of activity recorded), and collected points were redeemable for rewards (downloadable retail vouchers) sponsored by, and redeemable at, local businesses. To reduce the risk of ‘gaming’ (i.e. any items to cheat the system in order to gain more points without doing PA, for example, driving rather than walking between sensors), a daily points cap was implemented and the transit times between wifi beacons checked for anomalous values. Bonus rewards and Double Points Days were offered to participants.

Participants completed a Contingent Valuation (CV) survey [16] at baseline which provided their stated preferences to assess the minimum level of financial incentive necessary for them to increase moderate-vigorous physical activity (MVPA) (Additional file 2). This information helped determine the overall level of the rewards available for earned points. The intervention had several other components designed to enhance the effectiveness of the incentives. The techniques included the provision of regular tailored motivational emails, tailored feedback, and information on walking routes in the vicinity of the participating workplaces and links to other resources such as PA advice. It also included self-regulation techniques of goal setting, self-monitoring, and prompts to behaviour (described in detail elsewhere) [14]. These components were delivered via the study website and designed to have multiple effects: (a) to increase usage of the study website, (b) as effective behaviour change techniques in their own right, and (c) as techniques designed to aid the transition from more extrinsically motivated behaviour to more intrinsically motivated habitual behaviour.

The financial incentive component of the intervention was based on principles of Learning Theory [17] by providing an immediate reward (extrinsic motivation) for behaviours that offer health gains in the future. The use of contingent reinforcement hypothesised that repetition of the behaviour-reward cycle would lead to workplace PA becoming a ‘learned’ behaviour and lead to habit formation, which fit within a self-regulation control theory framework. Motivational messages (persuasion) and social support (vicarious experience) would increase self-efficacy satisfaction with the consequences of behaviour change acting as a reinforcing mechanism, in addition to the reinforcement of financial incentives. Thus, the financial incentive component was embedded in a complex intervention containing evidence-informed behaviour change techniques. A logic model underpinning the intervention was developed (Additional file 3).

Those assigned to the waiting-list control group (*n* = 396) were offered the opportunity to participate in the intervention after the final follow-up period (i.e. 12 months post-baseline). Participants in this group completed outcome measures at the same time points as the intervention group but received no other intervention.

### Outcomes

The primary outcome was mean steps/day (objectively measured via the validated Yamax Digi-walker CW-701 pedometer worn for seven consecutive days) for participants at the 6 month follow-up. This outcome was also collected at 12 month follow-up. Although only workplace PA was incentivised, we hypothesised that participants would be encouraged to be more active generally and so we chose a measure of total PA as our primary outcome which is most public health relevant.

Secondary outcomes at 6 month follow-up were self-reported measures of workplace PA (Global Physical Activity Questionnaire; GPAQ), health (Short Form-8), well-being (Warwick-Edinburgh Mental Well-being Scale; WEMWBS), health-related quality of life (EQ-5D-5 L), and work-related impacts (absenteeism and presenteeism using the WHO Health and Performance Questionnaire).

Hypothesised mediators of initiation and maintenance of PA were collected at baseline, at 4 weeks and 6 months post-baseline (the full list of measures is in Additional file 4).

Health-care service use and medication prescriptions were collected by self-report for the health economic evaluation.

Further details on outcome measures are provided in the Additional file 4). Self-reported outcome measures were collected at baseline and 6 months (unless otherwise stated), via online questionnaires distributed by email and automatically collated via Qualtrics (www.Qualtrics.com).

### Statistical analysis

This study was powered to detect a minimum difference in means of 30 min of MVPA/week between groups. This is equivalent to an effect size of 0.40 assuming the outcome has a SD of 75 min. A study of 330 per group (or 660 in total) would have 90% power at the 5% significance level to detect this difference with allowance made for an intra-cluster correlation coefficient of 0·029, an assumed mean cluster size of 20 and a coefficient of variation in cluster size of 1·0. Assuming a 15% drop-out, the study would therefore need to randomise 776 participants. This revised sample size was a change to protocol [14].

#### Primary and secondary analysis

Primary and secondary outcomes at six and 12 months post-baseline (where applicable) were compared between intervention and control groups using analysis of covariance (ANCOVAs) adjusting for baseline values, randomisation stratum (Large 50+ employees, Medium 20–50 employees, Small < 20 employees or Schools/Colleges) and season with standard errors (SEs) and *p*-values corrected for clustering. Analysis was by intention to treat. The impact of non-response on the primary outcome findings was investigated by multiple imputation.

#### Mediation analysis

Hypothesised mediators of initiation and maintenance of PA behaviour change were compared between intervention and control groups using random-effects regressions. Structural equation models (SEM) were also run for all mediators of initiation and maintenance with group assignment as the independent variable, the 4 week/6 month mediator as the mediating variable and 6 month pedometer steps/day as the dependent variable. All analyses were adjusted for baseline values of the mediator and outcome, randomisation stratum and season with SEs and p-values corrected for clustering (Additional file 5). This analysis was conducted to determine whether there was a significant relationship between the mediator and outcome when controlling for group assignment.

#### Economic evaluation

The primary economic evaluation took the form of a within-trial cost utility analysis, adopting a public sector perspective as recommended by the UK’s National Institute for Health and Care Excellence (NICE) [18] for interventions with health and non-health outcomes in the public sector (and other settings). Costs included the intervention costs (apportioned per participant) and health-care resource use. Health outcomes were expressed in terms of quality-adjusted life-years (QALYs) accrued over the 6 month follow-up period. The primary economic analysis reported an incremental cost-effectiveness ratio (ICER) estimated by dividing the adjusted difference in mean costs between groups by the adjusted difference in mean QALYs between groups. ICER estimates were compared with a £20,000 - £30,000 per QALY threshold applied by NICE. A supplementary Cost Benefit Analysis (CBA) was undertaken from an employer’s perspective by using a ‘net-cost model’, incorporating the intervention costs and the avoided costs of absenteeism and productivity loss due to sick days using a human capital approach (Additional file 6).

The level of significance was *p* < 0.05 for all statistical and economic analyses. Analyses were carried out using Stata release 13 (StataCorp, College Station, TX, USA).

A detailed process evaluation is published elsewhere [19, 20].

## Results

**Fig. 1:**
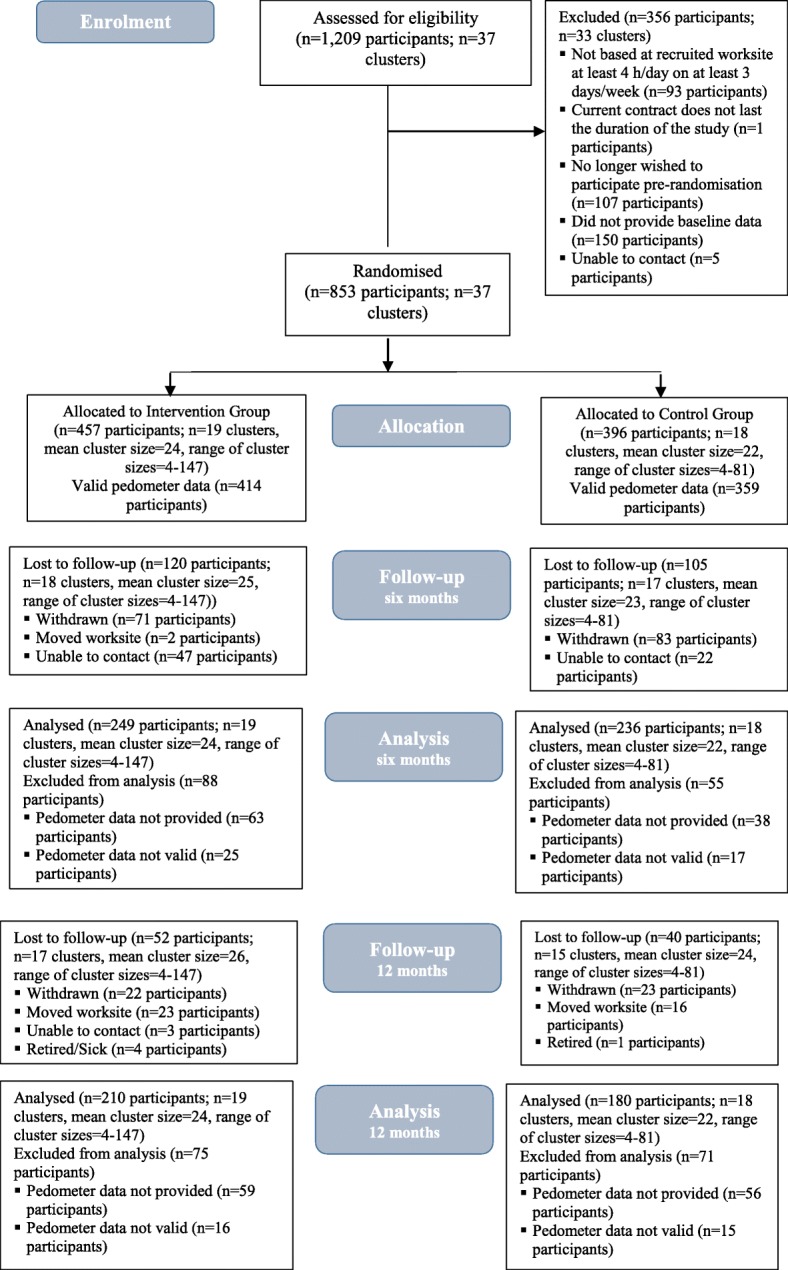
CONSORT diagram

Workplaces were recruited between September 2014 and August 2015 and participant recruitment took place between January 2015 and October 2015. A total of 1209 employees expressed an interest in participating in the study and were assessed for eligibility. Of these, 853 participants from 37 clusters were recruited and randomised into two groups (*n* = 457 intervention group, *n* = 396 control group). The number of clusters randomised per organisation ranged from 1 to 13, and the mean number of participants per cluster was 23 (Fig. 1). Participants redeemed 39% (SD 43%) of their earned points.

Table 1 shows baseline characteristics of the clusters and participants by group. The mean age of participants was 43·6 (standard deviation (SD) 9·6) years and 71% were female. At baseline, mean steps/day were 7826 (SD 3425).

**Table 1:** Mean (SD) baseline characteristics of participants according to group

	Intervention Group	Control Group
*Characteristics of clusters*	N = 19	N = 18
Number of participants; mean (range)	24 (4 to 147)	22 (4 to 81)
Randomisation stratum, clusters (n, % participants)		
Small (< 20 employees)	11 (114, 25%)	11 (105, 27%)
Medium (20–50 employees)	5 (167, 37%)	4 (123, 31%)
Large (> 50 employees)	1 (147, 32%)	2 (144, 36%)
Schools	2 (29, 6%)	1 (24, 6%)
*Characteristics of participants*	n = 457	*n* = 396
Age (years)	44·0 (9·3)	43·0 (10·0)
Female gender; n (%)	329 (72%)	278 (70%)
BMI (kg/m^2^)	27·2 (5·6)	26·6 (5·3)
Income >£20 k; n (%)	341 (75%)	291 (73%)
Education some higher level; n (%)	295 (65%)	270 (68%)
Marital status married/co-habiting; n (%)	313 (68%)	274 (69%)
Objective PA: pedometer steps (steps/day)	7977 (3602)	7650 (3204)
Objective: physical activity category, n (%)		
High (> 7500 steps/day)	204 (45%)	167 (42%)
Moderate (> 2500- ≤ 7500 steps/day)	199 (44%)	184 (46%)
Low (≤2500 steps/day)	11 (2%)	8 (2%)
GPAQ: minutes of work PA (minutes/week)	42 (138)	58 (151)
GPAQ: minutes of MVPA (minutes/week)	296 (342)	344 (333)
GPAQ: physical activity category^a^, n (%)		
High	70 (15%)	76 (19%)
Moderate	140 (31%)	130 (33%)
Low	141 (31%)	104 (26%)
SF-8: Mental Component Score	48·0 (8·9)	47·7 (9·3)
SF-8: Physical Component Score	52·5 (6·6)	52·7 (7·0)
EQ-5D: Health State	82·4 (13·8)	83·8 (14·3)
EQ-5D: Weighted Health Index	0·89 (0·11)	0·89 (0·12)
WEMWBS: Mental wellbeing scale	50·2 (8·2)	50·3 (8·9)
Physical Activity Self-Efficacy scale	2·91 (0·97)	2·92 (0·94)
HPQ: Four week absolute absenteeism	5·04 (41·3)	3·48 (50·0)
HPQ: Absolute presenteeism	80·3 (13·6)	81·0 (13·4)
HPQ: Combined relative absenteeism and absolute presenteeism	8·84 (12·57)	8·56 (7·32)

^a^Physical activity category was derived based on the standardised scoring protocol for the GPAQ (http://www.who.int/ncds/surveillance/steps/GPAQ%20Instrument%20and%20Analysis%20Guide%20v2.pdf)

*EQ-5D* EuroQol five dimensions, *GPAQ* Global Physical Activity Questionnaire, *HPQ* Health and Work Performance Questionnaire, *MVPA* Moderate- to vigorous-intensity physical activity, *NHS* National Health Service, *PA* Physical activity, *SD* Standard deviation, *SF* Short Form, *WEMWBS* Warwick-Edinburgh Mental Wellbeing Scale

Please see Additional file 4 : Table S1 for further details regarding the outcome measures

At the 6 month follow-up, all clusters remained in the trial and we analysed 249 (54·4%) of 457 participants in the intervention group and 236 (59·6%) of 396 participants in the control group for the primary outcome (Fig. 1).

At 6 month post-baseline, ANCOVAs showed there was a significantly reduced mean steps/day in the intervention group relative to the control group (adjusted *b* = − 336, 95% CI: -612 to − 60, *p* = 0·02 adjusted for season) (Table 2). Mean steps/day decreased (from baseline) by 947 steps (SD 2702) in the intervention group, and by 398 steps (SD 2471) in the control group. The analysis was repeated following imputation of missing data by chained equations (Additional file 7). The corresponding difference in mean steps/day was − 526 (95% CI: -948 to − 104, *p* = 0·02), in the same direction as the primary analysis.

**Table 2:** Mean (SD) primary outcome at 6 months and 12 months according to group and ANCOVA results before and after adjusting for season

Outcome	Intervention Group^a^		Control Group^a^		Analysis of covariance^b^		Analysis of covariance^c^	
	N	Mean (SD)	N	Mean (SD)	b (95% CI)	p-value	b (95% CI)	p-value
Six month: pedometer steps (steps/day)	249	6990 (3078)	236	7576 (3345)	− 519 (− 931, − 107)	0·01	− 336 (− 612, −60)	0·02
12 month: pedometer steps (steps/day)	210	7790 (3462)	180	8203 (3401)	− 561 (− 1243, 120)	0·11	−570 (− 1267, 127)	0·11

^a^Month six or month 12 outcomes (unadjusted)

^b^ANCOVA comparison of 6 month/12 month means in Intervention vs Control Group adjusted for baseline values of the outcome, and randomisation stratum and corrected for clustering

^c^ANCOVA comparison of 6 month/12 month means in Intervention vs Control Group adjusted for baseline values of the outcome, randomisation stratum and season and corrected for clustering

*ANCOVA* Analysis of covariance, *CI* confidence interval, *SD* standard deviation

At 12 months post-baseline, mean steps/day were 7790 (SD 3462) (decrease of 552 steps (SD 3183) from baseline) for the intervention group and 8203 (SD 3401) for the control group (increase of 98 steps (SD 2822) from baseline). ANCOVAs showed there was a non-significant difference between the intervention and control groups in steps/day (adjusted *b* = − 570, 95% CI: -1267 to 127, *p* = 0·11) (Table 2).

There were significant differences between groups for self-reported workplace PA (*b*-33·34, 95% CI: -65·44 to 1·24, p-0·04), with workplace PA scores higher in the control group at 6 months.

At 6 months, there was a significant difference between groups for the WEMWBS (*b* = 1·34, 95% CI: 0·48 to 2·20, *p* < 0·01) with wellbeing scores higher in the intervention group at 6 months. After adjusting for baseline values there were no significant differences between groups for SF-8 mental (*b* = 1·17, 95% CI: -0·23 to 2·56, *p* = 0·10) and physical (*b* = 0·64, 95% CI: -0·79 to 2·08, p = 0·38) component scores, the EQ-5D-5 L health state (*b* = 0·70, 95% CI: -2·45 to 3·86, p = 0·66) and weighted health index scores (*b* = 0·01, 95% CI: -0·01, 0·04, p = 0·37), and for 4 week absolute work absenteeism (*b* = − 2·59, 95% CI: -12·79 to 7·62, p = 0·62), and absolute presenteeism (*b* = 1·48, 95% CI: -0·43 to 3·38, p = 0·13) (Table 3).

**Table 3:** Mean (SD) secondary outcomes at 6 months according to group and ANCOVA results before and after adjusting for season

Outcome	Intervention Group^a^		Control Group^a^		Analysis of covariance^b^		Analysis of covariance^c^	
	n	Mean (SD)	n	Mean (SD)	b (95% CI)	p-value	b (95% CI)	p-value
GPAQ: minutes of work PA (minutes/week)	253	16 (58)	235	44 (129)	−34 (−65, −2	0.04	−33 (− 65, − 1)	0·04
GPAQ: minutes of MVPA (minutes/week)	231	291·8 (255·8)	221	350·1 (329·4)	−2·8 (− 63·4, 57·9)	0·93	4·1 (−47·1, 55·3)	0·88
SF-8: Mental Component Score	269	48·4 (8·9)	244	47·1 (9·4)	1·1 (−0·3, 2·4)	0·12	1·2 (− 0·2, 2·6)	0·10
SF-8: Physical Component Score	269	50·9 (8·4)	244	51·0 (7·6)	0·43 (−1·3, 2·1)	0·62	0·6 (−0·8, 2·1)	0·38
EQ-5D: Health State	262	78·0 (16·2)	239	78·3 (16·1)	0·7 (−2·4, 3·7)	0·66	0·70 (− 2·5, 3·9)	0·66
EQ-5D: Weighted Health Index	262	0·8 (0·1)	239	0·8 (0·2)	0·01 (−0·02, 0·03)	0·71	0·01 (−0·01, 0·04)	0·37
WEMWBS: Mental wellbeing scale	266	50·3 (8·4)	243	49·4 (8·2)	1·2 (0·2, 2·1)	0·02	1·3 (0·5, 2·2)	< 0·01
HPQ: Four week absolute absenteeism	247	4·0 (66·1)	227	7·0 (51·4)	−2·25 (−12·5, 8·0)	0·67	−2·59 (− 12·8, 7·6)	0·62
HPQ: Absolute presenteeism	261	78·6 (14·6)	236	78·5 (14·2)	0·8 (−1·8, 3·4)	0·54	1·5 (−0·4, 3·4)	0·13
HPQ: Combined relative absenteeism and absolute presenteeism	246	9·5 (24·6)	226	7·9 (4·9)	−0·03 (− 0·9, 0·9)	0·95	− 0·02 (− 0·9, 0·9)	0·96

^a^Month six outcomes (unadjusted)

^b^ANCOVA comparison of 6 month means in Intervention vs Control Group adjusted for baseline values of the outcome, and randomisation stratum and corrected for clustering

^c^ANCOVA comparison of 6 month means in Intervention vs Control Group adjusted for baseline values of the outcome, randomisation stratum and season and corrected for clustering

*ANCOVA* Analysis of Covariance, *CI* confidence interval, *EQ-5D* EuroQol five dimensions, *GPAQ* Global Physical Activity Questionnaire, *MVPA* moderate- to vigorous-intensity physical activity, *NHS* National Health Service, *PA* physical activity, *SD* standard deviation, *SE* standard error, *SF* Short Form, *WEMWBS* Warwick-Edinburgh Mental Wellbeing Scale

Table 3 shows the absolute absenteeism hours over a 4 week period for the intervention and control group was 4·04 (SD 66·11) and 7·01 (SD 51·40) respectively (*p* = 0·62). The difference was estimated to be 2·59 h (adjusted) over a 4 week period (p = 0·62). This equates to 15·54 h pro-rata for a 6 month time period. After attaching the hourly salary values, the avoided cost of absenteeism and the net cost of intervention for each representative salary grade were estimated. The adjusted cost saving for the employers ranged from £66–£735 depending on the wage rate employed. At current intervention cost (=£55·68), the probability that the intervention was cost-saving from the employer’s perspective was 64% for the high salary group, 62% for the middle salary group, and 57% for the low salary group (Additional file 8).

Table 4 shows the results of the effects on hypothesised mediators at 4 weeks and 6 months. Additional file 9 details baseline, week four and 6 month scores on mediator variables. At 4 weeks post-baseline, there were significant differences between the intervention and control groups for intentions (*b* = 0·29, SE = 0·13, *p* = 0·02), social norms (*b* = 0·23, SE = 0·08, *p* < 0·01), identified regulation (*b* = 0·14, SE = 0·06, p = 0·01), integrated regulation (*b* = 0·23, SE = 0·07, p < 0·01), and intrinsic motivation (*b* = 0·18, SE = 0·06, p < 0·01). There were non-significant between-group differences for the other hypothesised mediators on behaviour change at 4 weeks. There were no significant associations between mediator scores and 6 month pedometer steps/day in SEM models (Additional file 9). SRMR values were close to zero for all models, and CD values ranged from 0·56–0·76.

**Table 4:** Effects of intervention on hypothesised mediators, at 4 weeks or 4 months, with adjustment for baseline values (coefficients and standard errors from random-effects regressions)

MEDIATOR (range)	Four week mediators			Six month mediators		
	n	b (SE)	p-value	n	b (SE)	p-value
PA self-efficacy(1–5)	597	0·09 (0·08)	0·31			
Intentions (1–7)	595	0·29 (0·13)	0·02			
Outcome expectations (1–5)	528	−0·03 (0·06)	0·58			
Financial motivation (1–7)	600	0·11 (0·15)	0·46			
Planning (1–4)	575	0·02 (0·05)	0·75	436	0·08 (0·06)	0·18
Social norms (1–7)	576	0·23 (0·08)	< 0·01	434	0·10 (0·12)	0·42
Identified regulation (1–5)	598	0·14 (0·06)	0·01	459	0·11 (0·05)	0·02
Integrated regulation (1–5)	595	0·23 (0·07)	< 0·01	454	0·26 (0·08)	< 0·01
Intrinsic motivation (1–5)	599	0·18 (0·06)	< 0·01	456	0·17 (0·06)	< 0·01
Habit (1–5)				448	0·48 (0·12)	< 0·01
Workplace norms (1–5)				456	0·13 (0·07)	0·06
Recovery self-efficacy (1–4)				457	0·02 (0·06)	0·80
Maintenance self-efficacy (1–4)				459	−0·02 (0·09)	0·80
Outcome satisfaction (1–5)				427	0·03 (0·05)	0·56

*PA* Physical activity, *SE* Standard error

NB. All questionnaire items were scaled so that lower values indicated lower levels of the mediator/outcome. Results are adjusted for strata, season, baseline pedometer steps/day and baseline mediator values with cluster-adjusted standard errors and p-values (*b* = coefficient for group assignment variable, i.e. Intervention versus Control)

At 6 months, there were significant differences between the intervention and control groups for identified regulation (*b* = 0·11, SE = 0·05, p = 0·02), integrated regulation (*b* = 0·26, SE = 0·08, p < 0·01), intrinsic motivation (*b* = 0·17, SE = 0·06, p < 0·01), and habit (*b* = 0·48, SE = 0·12, p < 0·01). There were non-significant between-group differences for the other hypothesised mediators on behaviour change at 6 months.

Results of the single mediator models with 6 month pedometer steps/day (Additional file 10) showed significant, positive intervention effects on 4 week mediator scores for intentions, social norms, identified regulation, integrated regulation and intrinsic motivation. There were no significant associations between mediator scores and 6 month pedometer steps/day. Tests of the association between mediators and PA were significant and positive for planning, social norms, identified regulation, integrated regulation, intrinsic motivation and habit. In contrast, tests of the association between mediators and PA were significant and negative for workplace norms. SRMR values were close to zero for all models, and CD values ranged from 0·62–0·76 (Additional file 10).

The intervention costs and use of health-care resources and services are detailed in the Additional files 11 and 12. Overall, there were no statistically significant differences in the quantity of each resource item between the groups; the adjusted cost of healthcare resources per participant is £190·14 for the intervention group and £247·70 for the control group (*p* = 0·23). The cost utility results for the intervention are presented in Table 5. Overall, the intervention was approximately £26·00 per participant more costly but had no statistically significant effect on QALYs as compared to the control group. The average cost per participant was £253·49 (95% CI £188·41 to 318·57) in the intervention group and £227·64 (95% CI £170·86 to 284·43) in the control group. Mean QALYs accrued over the 6 months trial period were 0·4157 (95% CI 0·4077 to 0·4238) for the intervention group and 0·4158 (95% CI 0·4057 to 0·4260) for the control group, leading to a 0·0000891 (95% CI -0·008 to 0·008) lower QALY gain in the intervention group compared to the control group. A 1000 bootstrap uncertainty analysis revealed that the probability that the intervention was cost-effective at the £30,000 per QALY threshold was 34·6%.

**Table 5:** Cost-effectiveness results (incremental cost per QALY) with 6 month follow-up

Treatment group	Cost (£)^a^		QALY^b^	
	Mean	95% CI	Mean	95% CI
Intervention (n = 457)	253·49	188·41, 318·57	0·4157	0·4077, 0·4238
Control (n = 396)	227·64	170·86, 284·43	0·4158	0·4057, 0·4260
Difference (95% CI)	25·85	−29·89, 81·60	−0·0000891	−0·008, 0·008
ICER	-£ 290,178 per QALY			
95% CI for ICER (from bootstrap)	-£480,012 to -£100,336 per QALY			

^a^Adjusted cost, per-participant intervention cost included in the cost of intervention group

^b^Adjusted QALY

Costs were estimated using generalised linear models, gamma family, log link. Decrements of QALY were estimated using generalized linear models, gamma family, log link. Arm level costs and QALYs were obtained from recycled predictions. Covariates for the models were: baseline cost and baseline utility, mean steps, age, sex, SF-8 physical and mental scores, cluster, Strata, and season. 95%CIs were obtained from 1000-iteration bootstrap

*CI* Confidence interval, *ICER* Incremental cost-effectiveness ratio, *QALY* Quality Adjusted Life Year, *SE* Standard error

## Discussion

In this cluster RCT, there was a significant reduction in mean steps/day (the primary outcome) in the intervention group compared to the control group at 6 months post-baseline (adjusted mean difference = − 336, 95% CI: -612 to − 60, *p* = 0·02). There was no significant difference between the intervention group and the control group in mean steps/day at 12 months post-baseline (adjusted mean difference = − 570, 95% CI: -1267 to 127, p = 0·11).

By contrast, the study found significant intervention effects for improvement in mental wellbeing. However, this was not supported with findings from the quality of life and mental health measures, and so we are cautious in over-interpreting this positive finding. It is conceivable that mental wellbeing improved in the intervention group as a result of being involved in an employee health and wellbeing trial at a time when there were significant re-structuring in the organisations.

Further, there was a 60% probability of the intervention being cost-saving from an employer’s perspective arising from reduced absenteeism (p = 0·62). Whilst the decline in absenteeism hours was not statistically significant it is arguably economically significant and worth exploring in future studies powered to detect such economic impacts [21]. Hence, from the employers perspective and valuing absenteeism using a human capital approach, the intervention could be deemed to be worthwhile.

Our analyses included an adjustment for season which would suggest that the 6 month difference in steps/day between the intervention and control groups was not due to a seasonal effect. Given the rationale for intervention, its theoretical underpinning and our pilot data, these results defy easy explanation. Our qualitative findings point to some issues with the level and type of incentives [19]. Participants redeemed 39% (SD 43%) of their earned points. These aspects were informed a priori via a CV survey with all participants, and with the types of vouchers were discussed in pre-intervention focus groups with the target population. They were also shown to be popular in our pilot work [12]. Our intervention was 6 months in duration which is in line with other PA interventions attempting to elicit and support PA maintenance (a period consonant with the mean in a recent review [10]). Usage data from the PA monitoring system showed that the minutes of activity recorded on the system declined over the 6 month intervention period (participants logging at least 10 min of activity via the PA monitoring system on 25% of all possible intervention days). However, from our qualitative findings it is clear that some participants reported feeling frustrated with early technological glitches that impacted on accurate monitoring of PA behaviour [19, 20]. New rewards, walking routes, and double point’s days were regularly introduced in an attempt to keep the format fresh and appealing but this did not improve workplace PA. The importance of study context should not be underestimated in the interpretation of our study findings. During our recruitment phase, a number of participating organisations undertook significant re-structuring due to the then current economic austerity, resulting in uncertainty regarding job security and job location; a time when employee health and wellbeing was at its most vulnerable. The impact of this was evident in our qualitative data which highlighted how motivation can be more usefully seen as a property of systems (incorporating technologies, organisation and action) rather than just of individuals [19]. Further details regarding the findings from the process evaluation are provided in Gough et al. (2018) [19] and Murray et al. (2019) [20].

Our findings suggest interesting conjectures regarding causal mechanisms. The use of financial incentives to change health behaviours is often criticised for ‘crowding out’ intrinsic motivation in line with Self Determination Theory - albeit based on laboratory based experiments [22]. Findings from our ‘real-world’ study provide evidence to the contrary in two ways: (1) our results showed increased internal forms of motivation (i.e. identified regulation, integrated regulation and intrinsic motivation) for the intervention group compared to the control group at 4 weeks and 6 months; (2) results demonstrated that there was no intervention effect for financial (extrinsic) motivation at 4 weeks. These findings have important implications in respect of some of the contentious issues highlighted in the literature on the use of financial incentives for achieving behaviour change. They suggest that using financial incentives within a complex behaviour change intervention with multiple components collectively does not necessarily diminish, and may facilitate intrinsic motivation. Our results also suggest that the provision of financial incentives does not necessarily increase financial (extrinsic) motivation. These findings are in line with a systematic review of psychological and economic studies which concluded that there was no evidence that extrinsic incentives would crowd out incentivised health behaviours [23]. However, given the complex nature of this intervention, it is important to note that the positive findings for internal motivation could also be related to our use of self-regulation Behaviour Change Techniques including self-monitoring and feedback on PA behaviour.

More generally, whilst the intervention group showed increases in some hypothesised mediators of initiation, these increases were not related to PA behaviour at 6 months. However, hypothesised mediators of maintenance were related to PA behaviour at 6 months. Especially notable are the findings that internal motivation mitigated reduction in PA behaviour at 6 months. Self-regulation appears to have mitigated and attenuated the reduction in steps in the intervention group. As reported above, our results run counter to predictions of Self Determination Theory suggesting that there is a role for financial incentives and self-regulation interventions. However, the amount of variance explained by our measured mediators of behaviour change was low. Therefore, future studies would need to examine other potential mediators of behaviour change to shed further light on these associations. Our previous systematic review [11] suggested that studies of mechanisms of intervention effect have generally been of poor methodological quality and would benefit from a framework based on consensus about how mediation should be measured and tested in trials of complex interventions. Such a framework should include the use of formal mediation tests, the embedding of evidence-based techniques for changing hypothesised mediators and the need to investigate constructs with particular relevance for initiation *and* maintenance of behaviour change. Future research should also examine mediators of adverse effects such as found in the present study so we can better understand unintended consequences and negative findings [24].

Despite the improvements in some secondary outcomes and positive net-benefit for employers, the primary outcome (steps/day) declined significantly in the intervention group. These results pose several scientific and real world implementation challenges that are too infrequently exposed in public heath intervention trials [25], including how to balance positive and negative results when primary and secondary outcomes are discordant [26].

There is a long standing belief that positive results are favoured by scientific journals and that this may contribute to “publication bias”. On the other hand, some journals claim now to select articles for publication based on their contribution to the literature and welcome null results that challenge conventional wisdom or prior expectations [27]. The results from our trial certainly challenged prior expectations. However, it is notoriously hard to disprove any hypothesis, and so negative studies must have the precision and strength of design to be reasonably persuasive. The revised sample size and power calculations for our trial were approved by the study funder, and the effect size on which they were premised was within the category of “moderate” effect sizes observed in past PA interventions [28]. Even though attrition and loss to follow-up was higher than predicted (43% observed vs 15% predicted), (albeit comparable to literature published during the conduct of our trial.) [29] we might have concluded that accepting the null hypothesis at 12 months was justified. However, the primary outcome was specified at 6 months and the non-significant difference in mean steps/day at 12 months (~ 500 steps) was, arguably, still of a magnitude which could be of public health importance. We would probably have claimed as much had the direction of the intervention effect been positive. Some may also argue that with a primary outcome of total PA, the lack of a significant intervention effect is not surprising given that the intervention specifically incentivised workplace PA. However, we hypothesised that participants would also be encouraged to further participate in additional PA outside the workplace. Workplace PA was specifically measured using the remote sensing monitoring system (sensors and keyfobs) and the GPAQ which incorporates a workplace PA domain. However, we also used a well-validated pedometer, followed a standardised measurement protocol (including sealing the pedometer to prevent reactivity), and we supplemented the data with the GPAQ and daily PA monitoring using the remote sensing system. There was consistency in the direction and magnitude of our findings for total PA and workplace PA.

If the significant negative effect at 6 months is indicative of a true negative effect *of this intervention in this setting*, one might still ask whether the result is generalisable. Is this recruited sample of public sector office workers in Northern Ireland representative of office workers elsewhere in the UK? We have no reason to conclude that it is not [3, 5, 7, 13], but are mindful of the modelling undertaken by Basu and Kiernan [30] which demonstrated that two key factors impacting the success of workplace-based financial incentives for behaviour change are (i) *who* participates (this will be explored in a detailed process evaluation as the overall null effect might be masking differential effects in different population sub-groups) and (ii) the *levels* of incentives. Thus it is important to further investigate the potential causal mechanisms using mediation and moderation analyses to gain a better understanding of our findings. Our study was not powered to detect changes in mediating variables, and because of low power and multiple testing, the results need to be interpreted with caution.

## Conclusions

In summary, the PAL Scheme intervention was not more effective than waiting-list control. Reduced health care costs, reduced absenteeism and improved mental wellbeing in the intervention group are somewhat noteworthy, and results suggest that the intervention could be cost beneficial for employers. However this needs to be explored within a trial powered on the economic outcome of productivity and/or absenteeism. Finally, we believe our results pose several scientific and real world implementation challenges that are too infrequently exposed in public heath intervention trials.

## Additional file

Additional file 1: Components of the physical activity monitoring system (wifi beacons, keyfobs and website). (DOCX 1236 kb)

Additional file 2: Methodology: Contingent Valuation Survey to elicit plausible financial incentives required for increasing physical activity. (DOCX 20 kb)

Additional file 3: **Figure S1.** Logic model of the Physical Activity Loyalty scheme. (DOCX 62 kb)

Additional file 4: **Table S1.** Description of the primary and secondary outcomes and measures. (DOCX 27 kb)

Additional file 5: Methodology-Mediation analyses. (DOCX 21 kb)

Additional file 6: Methodology-Economic evaluation. (DOCX 23 kb)

Additional file 7: **Table S2.** Mean (SD) outcomes at months six and 12 according to group and ANCOVA results before and after adjusting for season, with imputation of missing values on the six or 12 month outcomes. (DOCX 20 kb)

Additional file 8: **Table S3.** Estimation of avoided cost of absenteeism and net cost of intervention using the ‘net cost model’. (DOCX 20 kb)

Additional file 9: **Table S4.** Baseline, four week and six month scores on mediator variables. (DOCX 23 kb)

Additional file 10: **Table S5.** Results of single mediator models with six month pedometer steps/day as the dependent variable. (DOCX 24 kb)

Additional file 11: **Table S6.** Intervention costs. (DOCX 21 kb)

Additional file 12: **Table S7.** NHS and social care resource use per participant over six months (complete case). (DOCX 24 kb)

## Abbreviations

ANCOVA: Analysis of Covariance
BMI: Body Mass Index
CBA: Cost Benefit Analysis
CD: Coefficient of Determination
CIs: Confidence Intervals
CV: Contingent Valuation
EQ-5D: Euroqol 5 dimension
EQ-5D-5 L: Euroqol 5 dimension, 5 level
GPAQ: Global Physical Activity Questionnaire
ICER: Incremental Cost-Effectiveness Ratio
ISRCTN: International Standard Randomised Controlled Trial Number
MVPA: Moderate-to-Vigorous Physical Activity
NHS: National Health Service
NICE: The National Institute for Health and Care Excellence
NIHR: National Institute for Health Research
ORECNI: Office of Research Ethics Committees Northern Ireland
PA: Physical Activity
PAL: Physical Activity Loyalty
PAR-Q: Physical Activity Readiness Questionnaire
PHR: Public Health Research
PSSRU: Personal Social Service Research Unit
QALY: Quality Adjusted Life Year
RCT: Randomised Controlled Trial
SD: Standard Deviation
SE: Standard Errors
SEM: Structural Equation Modelling
SF-8: Short Form 8
SMD: Standardised Mean Difference
SRMR: Standardised Root Mean Square Residual
WEMWBS: Warwick-Edinburgh Mental Wellbeing Scale
WHO: World Health Organization
