# Supplementary material for: Effectiveness and cost-effectiveness of a loyalty scheme for physical activity behaviour change maintenance: results from a cluster randomised controlled trial
Source: Int J Behav Nutr Phys Act. 2018 Dec 12;15:127. doi: 10.1186/s12966-018-0758-1 (PMC6291971; doi:10.1186/s12966-018-0758-1)

**Components of the physical activity monitoring system (wifi beacons, keyfobs and website)**

1.
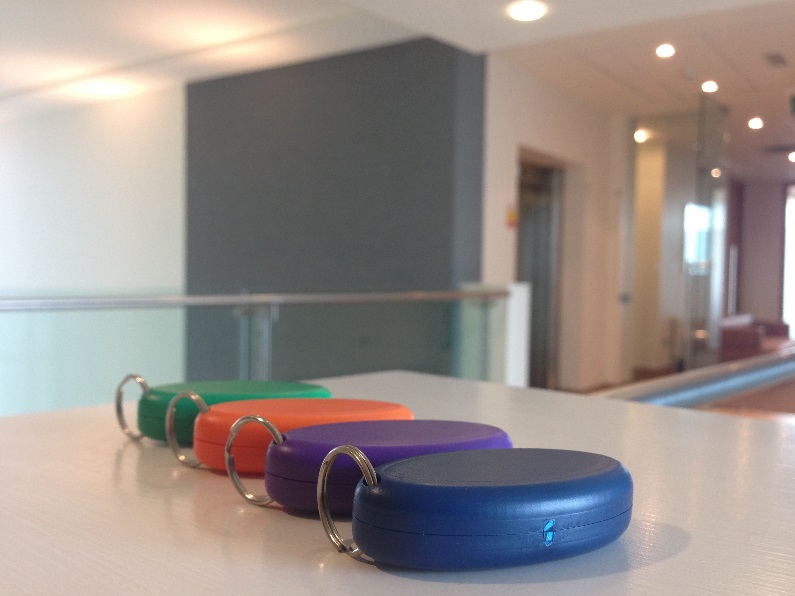
Wifi beacons

b) Keyfobs

c) Website


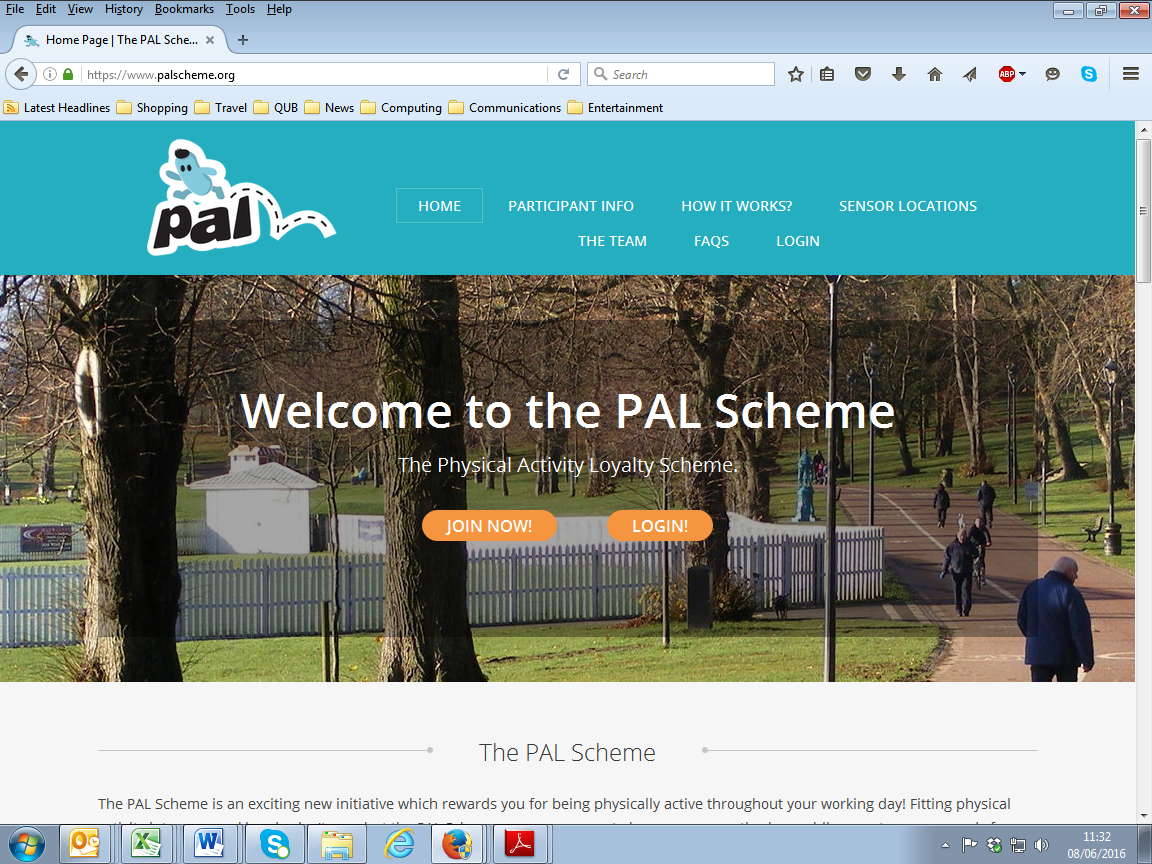

Supplement: Supplementary file 1 — Components of the physical activity monitoring system (wifi beacons, keyfobs and website). (DOCX 1236 kb) [file 12966_2018_758_MOESM1_ESM.docx]
