# Supplementary material for: Effectiveness and cost-effectiveness of a loyalty scheme for physical activity behaviour change maintenance: results from a cluster randomised controlled trial
Source: Int J Behav Nutr Phys Act. 2018 Dec 12;15:127. doi: 10.1186/s12966-018-0758-1 (PMC6291971; doi:10.1186/s12966-018-0758-1)
Supplement: Supplementary file 2 — Methodology: Contingent Valuation Survey to elicit plausible financial incentives required for increasing physical activity. (DOCX 20 kb) [file 12966_2018_758_MOESM2_ESM.docx]

**Methodology: Contingent Valuation survey to elicit plausible financial incentives required for increasing physical activity**

To investigate the optimal levels of financial incentives required to encourage changes in physical activity (PA), participants in the PAL study were sent an online questionnaire at baseline to assess the average level of incentives that participants would be Willing-to-accept (WTA) to increase their overall PA level. Contingent Valuation, a survey-based economic technique known in the valuation of potential future or hypothetical (but realistic) non-market goods and interventions, was used.^1^ The values obtained in the preliminary Contingent Valuation analysis were later used to set the level of the rewards available for earned “points”. Through an online questionnaire, participants were asked to answer two dichotomous hypothetical questions which asked respondents to state their willingness to participate in the scheme if certain financial incentives were provided. To investigate whether the minimum monetary incentives required by participants varied across the range of proposed PA increments, we considered two separate scenarios, i.e. an increase of 30 or 60 minutes of general PA per week. Further, to avoid order effects, participants were randomised into two groups, with one group first presented with the 30-minute PA scenario and then the 60-minute PA scenario; and the other group presented first with 60-minute PA scenario and then the 30-minute PA scenario.

Since what was a reasonable bid set was unknown, we provisionally suggested some initial bid sets which were pre-tested among a pilot group of 98 participants. Each participant was presented with a starting bid level randomly-chosen from four levels, i.e. £0.50, £1, £3.00, and £5.00, for the 30-minute PA scenario and £0.75, £1.50, £5.00, and £7.50, for the 60-minute PA scenario. Since more than half of the participants indicated “Yes” even for the smallest bids, the bids were replaced by smaller bids. The bid sets were revised as £0.10, £0.50, £1.50, and £5.00 for the 30-minute PA scenario and £0.15, £1.00, £3.00, and £10.00 for the 60-minute PA scenario. An updated questionnaire with the new bids was then sent to another 74 participants. The range of the updated bid levels covers the WTA distribution well because the highest bid level causes approximately 90% of “Yes” responses whereas the smallest bid levels result in approximately 10% of “Yes” responses. The non-parametric Turnbull method^2^ was used to estimate WTA based on responses of the 172 participants.

Results showed that, on average, the plausible levels of financial incentives required by participants was £1.24 for increasing general PA for 30 minutes (4.1p/minute) and was £1.95 per week for increasing 60 minutes per week (3.2 p/minute), respectively.

**References**

1. Carson RT. Contingent valuation: A user’s guide. Environmental Science and Technology 2000; 34: 1413-1418.

2. Haab TC and McConnell KE. Valuing environmental resources and natural resources: The econometrics of non-market valuation. Edward Elgar Publishing Limited, UK, 2002.
