# Supplementary material for: Effectiveness and cost-effectiveness of a loyalty scheme for physical activity behaviour change maintenance: results from a cluster randomised controlled trial
Source: Int J Behav Nutr Phys Act. 2018 Dec 12;15:127. doi: 10.1186/s12966-018-0758-1 (PMC6291971; doi:10.1186/s12966-018-0758-1)
Supplement: Supplementary file 3 — Figure S1. Logic model of the Physical Activity Loyalty scheme. (DOCX 62 kb) [file 12966_2018_758_MOESM3_ESM.docx]

#### Figure S1: Logic model of the Physical Activity Loyalty scheme

**Medium Term (12 mths)**

**Short-Term (6 mths)**

**Short-Term (0-6 mths)**

**Possible Mediators**

**Intervention**

**Long-Term (18 mths)**

Maintained behaviour change leads to maintained levels of:

Physical activity

Health

Mental wellbeing

Work absenteeism and presenteeism

Habit

Recovery self-efficacy

Social norms

Satisfaction with outcome expectancies

Further maintained behaviour change (> 3 yrs) leads to reduced risk of:

CVD

Cancer

Hypertension

Diabetes

Stroke

Multi-component intervention includes provision of points and rewards (non-cash financial incentives) contingent on meeting targeted behaviour goals

The following BCTs are included:

-immediate reward contingent on behaviour change; self-monitoring and feedback; info where/when to perform physical activity; specific goal setting; prompts and cues; action planning; barrier identification; social support; provision of info about health benefits of physical activity; habit formation; behavioural practice/rehearsal; behaviour substitution; adding objects to the environment; problem solving.

Gradual transition from focus on extrinsic motivation (rewards) to intrinsic motivation to increase physical activity by:

-reducing the extrinsic motivator by reducing the frequency of rewards given and rewards of lesser value;

-increase intrinsic motivators by increasing the emphasis on other BCTs in scheme, e.g. self-monitoring, feedback, goal setting, social support, planning, prompts and cues

Leads to increase in:

Physical activity

Health

Mental wellbeing

Work absenteeism and presenteeism

Recovery self-efficacy

Social norms

Perceptions of environment

Internet confidence

Outcome expectancy

Habit

Maintained behaviour change leads to:

-maintained increase of physical activity levels

**Uptake and Initiation:**

-self-efficacy

-intention

-outcome expectancies

-social norms

-discounting behaviour

-perceptions of workplace environment

-access to physical activity opportunities

-usage of PAL card

-usage of website

-web engagement and confidence

**Maintenance**

-habit

-recovery self-efficacy

-social norms

-satisfaction with outcome expectancies

**Underpinning Theoretical Framework**

Learning Theory

Social Cognitive Theory
