# Supplementary material for: Effectiveness and cost-effectiveness of a loyalty scheme for physical activity behaviour change maintenance: results from a cluster randomised controlled trial
Source: Int J Behav Nutr Phys Act. 2018 Dec 12;15:127. doi: 10.1186/s12966-018-0758-1 (PMC6291971; doi:10.1186/s12966-018-0758-1)
Supplement: Supplementary file 4 — Table S1. Description of the primary and secondary outcomes and measures. (DOCX 27 kb) [file 12966_2018_758_MOESM4_ESM.docx]

#### Table S1: Description of the primary and secondary outcomes and measures

| **Outcomes** | **Measures** |
| --- | --- |
| *Screening questionnaire* | |
| **Physical Activity Readiness Questionnaire (PAR-Q)** | Seven item self-administered screening questionnaire completed prior to participating in a physical activity intervention.^1^ |
| *Primary outcome* | |
| **Physical Activity**  Objectively measured via pedometer | The primary outcome was mean steps/day objectively measured by a sealed pedometer (to blind participants to the output) worn on the waistband (Yamax Digiwalker CW-701, Japan), for which reliability and validity has been established.^2-4^ Participants wore the pedometer for seven consecutive days on the waistband (dominant hip). They were asked to complete a wear time diary providing details on the dates/times that they removed the monitor and wore the monitor. Participants were advised to remove the monitor when showering, bathing or undertaking any water-based activities. Pedometer data was considered valid if the participant provided ≥250 steps/day for three or more days at each data collection period. Pedometers were sealed at all times to blind participants to the output and prevent reactivity which is standard practice. This device was used as a measurement tool only (i.e. was not part of the intervention) and a standardised measurement protocol used for the intervention and control group. We followed a standard protocol that has been well-validated and successfully employed as a measurement tool in numerous intervention studies. These data were collected at baseline, six months (immediately post-intervention) and 12 months (six months post-intervention). This schedule allowed us to account for seasonality of PA behaviours.  There are some limitations regarding the use of a pedometer for measuring our primary outcome. These include the inability to consider wear time in the processing of the data. The use of <250 steps/day as an indicator of non-wear could have removed participants who were inactive, or included participants who wore the monitor for a short period and erroneously classified them as inactive. The use of a pedometer meant we were not able to detect when in the day the PA was accumulated. However, we were able to specifically capture workplace PA using the remote sensing monitoring system (sensors and keyfobs) and the GPAQ which incorporates a workplace PA domain. However, we used a well-validated pedometer, followed a standardised measurement protocol (including sealing the pedometer to prevent reactivity), and we supplemented the data with the GPAQ and daily PA monitoring using the remote sensing system. |
| *Secondary outcomes* | |
| **Physical Activity**  Self-report | Participants were asked to complete the GPAQ to elucidate the context of PA undertaken.^5^ This was a paper-based questionnaire and participants were asked to complete this questionnaire immediately after the end of the seven day period of wearing the pedometer at baseline, six months and 12 months.  Variables for minutes of workplace PA (minutes/week), minutes of MVPA (minutes/week) and PA levels categorised as low, moderate and high were derived using the standardised GPAQ scoring protocol (<http://www.who.int/ncds/surveillance/steps/GPAQ%20Instrument%20and%20Analysis%20Guide%20v2.pdf>). |
| **Health and Wellbeing**  General health, mental health, quality of life and mental wellbeing | The following validated self-report measures were completed at baseline and six months via Qualtrics.  SF-8^6^: Items from the SF-8 questionnaire can be derived to give an indication of both physical and mental health.  EQ-5D-5L^7^: This is a measure of quality of life and used to derive the health state utility measure based on five dimensions of mobility, self-care, usual activities, pain/discomfort, and anxiety/depression (0-100), and the weighted health index. The EQ-5D-5L questionnaire is based on 5 dimensions of mobility, self-care, usual activities, pain/discomfort, and anxiety/depression, and a visual analogue scale (0-100) that assesses the participants’ health state.  WEMWBS^8,9^: derived from 14 statements (with higher scores indicating better mental health). The WEMWBS comprises 14 positively worded statements, where scores are summed with higher scores indicating greater mental well-being. |
| **Work-related Impacts**  Absenteeism and presenteeism | WHO HPQ^10^:  Work absenteeism was measured by asking participants to state the number of day’s sick leave in the past six months (collected at baseline and six months).  Specific questions from the WHO HPQ were used to measure work presenteeism. This validated method comprises three questions with answers on an 11-point Likert scale asking participants to rate their job performance levels. |
| **Mediators and Moderators**  Proposed mediators of PA behaviour change | Common core theoretical constructs of PA behaviour change included outcome expectancy,^12^  social norms,^12^  PA self-efficacy,^13^  financial motivation,^14^ planning,^15^ self-determined motivation (i.e. identified regulation, integrated regulation, intrinsic motivation)^16,17^ and intentions,^18^ were collected at baseline and four weeks to assess initiation of behaviour change.  Individual level, cognitive constructs measured to assess maintenance of PA behaviour change were collected at baseline and six months and included social norms and workplace norms,^12^ planning,^15^ self-determined motivation (i.e. identified regulation, integrated regulation and intrinsic motivation),^16,17^ habit,^19^ recovery and maintenance self-efficacy,^20^ and outcome satisfaction.^11,21^ |
| **Health Economic Evaluation** | Changes in HrQoL (as expressed using QALYs using EQ-5D-5L data) were measured from the participant’s perspective. The EQ-5D-5L is a validated measure and has been used extensively for cost-effectiveness analyses.  Utilisation of healthcare resources was captured using a specially devised online health and social care resource use data collection form.  These measures were completed at baseline and six months. Intervention costs were obtained using a modified template, explicitly discriminating between intervention and research costs. Briefly these include, website development, software (e.g. license fee), hardware (e.g. sensors, loyalty cards), and intervention running costs (e.g. maintenance of sensors), the costs of negotiating incentives from local businesses, and the delivery of vouchers. |

BMI: Body Mass Index; EQ-5D: Euroqol 5 dimensions; FFQ: Food Frequency Questionnaire; GPAQ: Global Physical Activity Questionnaire; HPQ: Health Work Performance Questionnaire; HrQoL; Health-related Quality of Life; PA: Physical activity; QALY: Quality Adjusted Life Year; SF-8: Short Form-8; WEMWBS: Warwick-Edinburgh Mental Wellbeing Scale; WHO: World Health Organisation

**References:**

1. Adams R. Revised Physical Activity Readiness Questionnaire. *Can Fam Physician.* 1999;45:992, 995, 1004-1005.
2. Bassett DR, Ainsworth BE, Leggett SR, Mathien CA, Main JA, Hunter DC, Duncan GE. Accuracy of five electronic pedometers for measuring distance walked. Med Sci Sports Exerc. 1996;28:1071–7.
3. Bravata DM, Smith-Spangler C, Sundaram V, Gienger AL, Lin N, Lewis R, Stave CD, Olkin I, Sirard JR. Using pedometers to increase physical activity and improve health: a systematic review. JAMA. 2007;298:2296–304.
4. Schneider PL, Crouter SE, Lukajic O, Bassett DR. Accuracy and reliability of 10 pedometers for measuring steps over a 400-m walk. Med Sci Sports Exerc. 2003;35:1779–84.
5. Bull FC, Maslin TS, Armstrong T. Global physical activity questionnaire (GPAQ): nine country reliability and validity study. *J. Phys. Act. Health* 2009;6(6):790-804.
6. Ware JE, Kosinski M, Dewey JE, Gandek B. *How to Score and Interpret Single-Item Health Status Measures: A Manual for Users of the of the SF-8 Health Survey*. Lincoln RI: QualityMetric Incorporated; 2001.
7. EuroQol Group. EuroQol: a new facility for the measurement of health-related quality of life. *Health Policy (New. York).* 1991;16:199-208.
8. Tennant R, Hiller L, Fishwick R, et al. The Warwick-Edinburgh Mental Well-being Scale (WEMWBS): development and UK validation. *Health Qual. Life Outcomes* 2007;5(1):63.
9. Lloyd K, Devine P. Psychometric properties of the Warwick–Edinburgh Mental Well-being Scale (WEMWBS) in Northern Ireland. *J. Ment. Heal.* 2012;21(3):257-263.
10. Kessler RC, Barber C, Beck A, et al. The World Health Organization Health and Work Performance Questionnaire (HPQ). *J. Occup. Environ. Med.* 2003;45(2):156-174.
11. Finch EA, Linde JA, Jeffery RW, Rothman AJ, King CM, Levy RL. The effects of outcome expectations and satisfaction on weight loss and maintenance: correlational and experimental analyses--a randomized trial. *Heal. Psychol.* 2005;24(6):608-616.
12. Ball K, Jeffery RW, Abbott G, McNaughton SA, Crawford D. Is healthy behavior contagious: associations of social norms with physical activity and healthy eating. *Int. J. Behav. Nutr. Phys. Act.* 2010;7(1):86.
13. Marcus BH, Selby VC, Niaura RS, Rossi JS. Self-efficacy and the stages of exercise behavior change. *Res. Q. Exerc. Sport* 1992;63(1):60-66.
14. Moller AC, Buscemi J, McFadden HG, Hedeker D, Spring B. Financial motivation undermines potential enjoyment in an intensive diet and activity intervention. *J. Behav. Med.* 2014;37(5):819-827.
15. Sniehotta FF, Schwarzer R, Scholz U, Schüz B. Action planning and coping planning for long‐term lifestyle change: theory and assessment. *Eur. J. Soc. Psychol.* 2005;35(4):565-576.
16. Markland D, Tobin V. A modification to the behavioural regulation in exercise questionnaire to include an assessment of amotivation. *J. Sport Exerc. Psychol.* 2004;26(2):191-196.
17. Wilson PM, Rodgers WM, Loitz CC, Scime G. “It’s who I am … Really!’ The importance of integrated regulation in exercise contexts. *J. Appl. Biobehav. Res.* 2007;11(2):79-104.
18. Fishbein M, Ajzen I. *Belief, Attitude, Intention and Behavior: An Introduction to Theory and Research*. Reading,Addison-Wesley; 1975.
19. Verplanken B, Orbell S. Reflections on past behavior: a self-report index of habit strength. *J. Appl. Soc. Psychol.* 2003;33(6):1313-1330.
20. Scholz U, Sniehotta FF, Schwarzer R. Predicting physical exercise in cardiac rehabilitation: the role of phase-specific self-efficacy beliefs. *J. Sport Exerc. Psychol.* 2005;27(2):135-151.
21. Rothman AJ, Sheeran P, Wood W. Reflective and automatic processes in the initiation and maintenance of dietary change. *Ann. Behav. Med.* 2009;38 Suppl 1:S4-17.
