# Supplementary material for: Effectiveness and cost-effectiveness of a loyalty scheme for physical activity behaviour change maintenance: results from a cluster randomised controlled trial
Source: Int J Behav Nutr Phys Act. 2018 Dec 12;15:127. doi: 10.1186/s12966-018-0758-1 (PMC6291971; doi:10.1186/s12966-018-0758-1)
Supplement: Supplementary file 5 — Methodology-Mediation analyses. (DOCX 21 kb) [file 12966_2018_758_MOESM5_ESM.docx]

**Methodology: Mediation analyses**

Single mediator models were run for all mediators of initiation and maintenance using the structural equation modelling (SEM) based product-of-coefficients approach^1^. In each model, the independent variable was group assignment, the mediating variable was the follow-up (i.e. four week or six month) mediator score, and the dependent variable was six month pedometer steps/day. Analyses were adjusted for randomisation stratum, season, baseline values of the mediator and baseline pedometer steps/day, with standard errors (SEs) and p-values corrected for clustering. Results are reported for tests of intervention effects on hypothesised mediators and tests of the association of hypothesised mediators with physical activity (PA). The maximum-likelihood (ML) method of estimation was used. Model fit was assessed using the coefficient of determination (CD), and standardised root mean square residual (SRMR) (≤0·08)^2^ which are reported with SEM models adjusting SEs and p-values for clustering in Stata.

For single mediator models, including mediators measured at four weeks and pedometer steps/day measured at six months (Appendix p15), there was a significant, positive intervention effect on four week mediator scores for intentions (*a*=0·39, SE=0·18, p=0·03), social norms (*a*=0·24, SE=0·09, p<0·01), identified regulation (*a*=0·14, SE=0·06, p=0·03), integrated regulation (*a*=0·22, SE=0·07, p<0·01) and intrinsic motivation (*a*=0·16, SE=0·06, p<0·01). There were no significant associations between mediator scores and six month pedometer steps/day. SRMR values were close to zero for all models, and CD values ranged from 0·56-0·76.

For single mediator models including mediators measured at six months and pedometer steps/day measured at six months (Appendix p15), there was a significant, positive intervention effect for habit (*a*=0·41, SE=0·14, p<0·01). Tests of the association between mediators and PA were significant and positive for planning (*b*=547, SE=180, p<0·01), social norms (*b*=262, SE=94, p<0·01), identified regulation (*b*=550, SE=211, p<0·01), integrated regulation (*b*=571, SE=188, p<0·01), intrinsic motivation (*b*=456, SE=176, p=0·01) and habit (*b*=482, SE=105, p<0·01). In contrast, tests of the association between mediators and PA were significant and negative for workplace norms (*b*=-362, SE=158, p=0·02). SRMR values were close to zero for all models, and CD values ranged from 0·62-0·76. Thus, whilst the total intervention effect on pedometer steps/day at six months was negative, this may have been partially mitigated by increases in planning, social norms, identified regulation, integrated regulation, intrinsic motivation and habit (baseline-six months), implying that participants experienced less of a decline in PA when they increased their levels of these constructs.

**References:**

1. Preacher KJ, Hayes AF. Asymptotic and resampling strategies for assessing and comparing indirect effects in multiple mediator models. *Behav Res Methods*. 2008;40(3):879-891.

2. Hooper D, Coughlan J, Mullen M. Structural equation modelling: guidelines for determining model fit. *Electron J Bus Res Methods*. 2008;6(1):53-60.
