# Supplementary material for: Effectiveness and cost-effectiveness of a loyalty scheme for physical activity behaviour change maintenance: results from a cluster randomised controlled trial
Source: Int J Behav Nutr Phys Act. 2018 Dec 12;15:127. doi: 10.1186/s12966-018-0758-1 (PMC6291971; doi:10.1186/s12966-018-0758-1)
Supplement: Supplementary file 6 — Methodology-Economic evaluation. (DOCX 23 kb) [file 12966_2018_758_MOESM6_ESM.docx]

**Methodology: Economic evaluation**

The economic evaluation takes the form of a within trial cost-utility (CUA) and cost-benefit analysis (CBA), comparing the PAL intervention group with the wait-list control group i.e. among the public sector employees in office-based settings. In line with the National Institute of Health and Care Excellence (NICE) recommendations for the conduct of economic evaluations of public health interventions the CUA analysis adopted a public sector perspective.^1^

Within trial costs include intervention costs (apportioned per participant) and health and social care resource use. Resource use data were collected from trial participants via an online questionnaire at baseline and 6 months. Service use data measured included visits to: GP, nurse, physiotherapist, A&E attendance, outpatient appointment, inpatient stays, medications use, and social care services including home care, meals on wheels, etc. Unit costs in the financial year 2015/2016 published by the Personal Social Service Research Unit (PSSRU)^2^ and NHS reference costs (where information not available from PSSRU^3^) were attached to each item of resource use.

QALYs were estimated using the utility index values generated from the EQ-5D-5L questionnaire collected from participants at baseline and six months follow-up. EQ-5D-5L responses from the participants were converted to utility scores by mapping the 5L descriptive system data onto the 3L valuation set, using the mapping algorithm developed by van Hout et al. (2012) as recommended by NICE guideline and its latest position statement published in August 2017.^4^ The area under the curve method was used to estimate QALYs over a 12-month period, following the trapeziums rule assuming a linear change in utility between each assessment time point.^5^

Results are presented using an incremental cost-effectiveness ratio (ICER) estimated by dividing the adjusted difference in mean costs between arms by the adjusted difference in mean QALYs between arms. Multiple imputation with chained equations was conducted for missing utility values and total costs at aggregate level. Intervention and control groups were imputed separately. Differences in mean cost and QALYs between the two groups were estimated using generalised linear models (GLM) which takes into account the typically skewed nature of cost and QALY data. Where histogram evidence identifies cost data as zero-inflated right-skewed, a gamma distribution with log links is recommended. When QALY data are in a distribution with a left-skewed tail bounded by 0·5 (maximum QALY accrued for 6 months period), decrements of QALYs are predicted in the GLM regression with a gamma family and log link. ICER estimates were compared with a £20,000 - £30,000 per QALY threshold applied by NICE [1]. A 1000-iteration Bootstrapping procedure was conducted to investigate the uncertainty surrounding the ICER estimate and the probability that the intervention was cost-effective under a wide range of hypothetical threshold (£0 - £100,000). Standard errors for differences in cost and QALYs were estimated through the bootstrap, adjusting for clusters.

In addition, a CBA was undertaken from an employer’s perspective by employing a ‘net-cost model’^6^ incorporating not only the intervention cost but also the avoided costs of absenteeism due to sick days. The WHO health and work performance questionnaire^7^ was used to capture number of hours absent from work, which was completed by participants at baseline and six months. Impacts on employee absolute absenteeism were measured using question four in the questionnaire: ‘About how many hours altogether did you work in the past four weeks (28 days)?’ and question two ‘How many hours does your employer expect you to work in a typical 7-day week?’, using the formula “4*Q2 – Q4”.

Individual hourly salary values in the financial year 2016 were attached to the number of hours absent from work. The hourly salary was obtained from NHS pay scales 2016, with the lowest grade at Band 1 (£7·80, equivalent to £15,251 annually), mid-grade at Band 8A (£22·86, equivalent to £44,703 annually), and highest grade at Band 9 (£50·85, equivalent to £99,437) taken separately to reflect the range of potential cost savings for employees at various salary grades. All analyses were undertaken according to the principle of intention-to-treat and in STATA/SE 12·0 (StataCorp, College Station, TX, USA).

**References:**

1. National Institute for Health and Care Excellence (2012). Methods for the development of NICE public health guidance (third edition) [PMG4]. 6 Incorporating health economics. [https://www.nice.org.uk/process/pmg4/chapter/incorporating-health-economics. Accessed 29 November 2017](https://www.nice.org.uk/process/pmg4/chapter/incorporating-health-economics.%20Accessed%2029%20November%202017).

2. Curtis, L., & Burns, A. (2016). Unit Costs of Health and Social Care. Canterbury: PSSRU, University of Kent at Canterbury.

3. NHS (2016). National Health Service Reference Costs. www.dh.gov.uk.

4. National Institute for Health and Care Excellence (2013). Guide to the methods of technology appraisal 2013. https://www.nice.org.uk/process/pmg9/chapter/the-reference-case#measuring-and-valuing-health-effects. Accessed 30 November 2017.

5. Brazier, J., Ratcliffe, J., Salomon, J. A., & Tsuchiya, A. (2017). Measuring and valuing health benefits for economic evaluation. Oxford: Oxford University Press Inc.

6. Lahiri, S., & Faghri, P. D. (2012). Cost-effectiveness of a workplace-based incentivized weight loss program. J Occup Environ Med, 54(3), 371-377.

7. Kessler, R. C., Barber, C., Beck, A., Berglund, P., Cleary, P. D., McKenas, D., et al. (2003). The World Health Organization Health and Work Performance Questionnaire (HPQ). J Occup Environ Med, 45(2), 156-174.
