# Supplementary material for: Effectiveness and cost-effectiveness of a loyalty scheme for physical activity behaviour change maintenance: results from a cluster randomised controlled trial
Source: Int J Behav Nutr Phys Act. 2018 Dec 12;15:127. doi: 10.1186/s12966-018-0758-1 (PMC6291971; doi:10.1186/s12966-018-0758-1)
Supplement: Supplementary file 7 — Table S2. Mean (SD) outcomes at months six and 12 according to group and ANCOVA results before and after adjusting for season, with imputation of missing values on the six or 12 month outcomes. (DOCX 20 kb) [file 12966_2018_758_MOESM7_ESM.docx]

**Table S2: Mean (SD) outcomes at months six and 12 according to group and ANCOVA results before and after adjusting for season, with imputation of missing values on the six or 12 month outcomes**

| **Outcome** | **Intervention Group^a^** | | **Control Group^a^** | | **Analysis of covariance^b^** | | **Analysis of covariance^c^** | |
| --- | --- | --- | --- | --- | --- | --- | --- | --- |
|  | **N** | **Mean**  **(SD)** | **N** | **Mean**  **(SD)** | **b**  **(95% CI)** | **p-value** | **b**  **(95% CI)** | **p-value** |
| Six month objective PA: pedometer steps (steps/day) | 457 | 6,914  (3,142) | 369 | 7,408  (3,253) | -574  (-1,109, -41) | 0.04 | -526  (-948, -104) | 0·02 |
| 12 month objective PA: pedometer steps (steps/day) | 457 | 7,522  (3,489) | 369 | 7,739  (3,340) | -543  (-1,207, 120) | 0.11 | -558  (-1,203, 87) | 0·09 |

^a^Month six or month 12 outcomes (unadjusted)

^b^ANCOVA comparison of six month/12 month means in Intervention vs Control Group adjusted for baseline values of the outcome, and randomisation stratum and corrected for clustering

^c^ANCOVA comparison of six month/12 month means in Intervention vs Control Group adjusted for baseline values of the outcome, randomisation stratum and season and corrected for clustering.

ANCOVA, Analysis of covariance; CI, confidence interval; EQ5D, EuroQol, five dimensions; GPAQ, Global Physical Activity Questionnaire; MVPA, moderate- to vigorous-intensity physical activity; NHS, National Health Service, PA, physical activity; SD, standard deviation; SE, standard error; SF, short form; WEMWBS, Warwick-Edinburgh Mental Wellbeing Scale
