# Supplementary material for: Effectiveness and cost-effectiveness of a loyalty scheme for physical activity behaviour change maintenance: results from a cluster randomised controlled trial
Source: Int J Behav Nutr Phys Act. 2018 Dec 12;15:127. doi: 10.1186/s12966-018-0758-1 (PMC6291971; doi:10.1186/s12966-018-0758-1)
Supplement: Supplementary file 8 — Table S3. Estimation of avoided cost of absenteeism and net cost of intervention using the ‘net cost model’. (DOCX 20 kb) [file 12966_2018_758_MOESM8_ESM.docx]

#### Table S3: Estimation of avoided cost of absenteeism and net cost of intervention using the ‘net cost model’

| **Grade** | **Avoided cost of absenteeism (£)** | **Net cost of intervention (Intervention cost minus the avoided cost of absenteeism)** |
| --- | --- | --- |
| Lowest (NHS Band 1, £7.8/hr) | £121·21 | -£65·53 |
| Mid (NHS Band 8A, £22.86/hr | £355·24 | -£299·56 |
| Highest (NHS Band 9, £50.85/hr) | £790·21 | -£734·53 |

Hr: Hour; NHS: National Health Service
