# Supplementary material for: Effectiveness and cost-effectiveness of a loyalty scheme for physical activity behaviour change maintenance: results from a cluster randomised controlled trial
Source: Int J Behav Nutr Phys Act. 2018 Dec 12;15:127. doi: 10.1186/s12966-018-0758-1 (PMC6291971; doi:10.1186/s12966-018-0758-1)
Supplement: Supplementary file 9 — Table S4. Baseline, four week and six month scores on mediator variables. (DOCX 23 kb) [file 12966_2018_758_MOESM9_ESM.docx]

#### Table S4: Baseline, four week and six month scores on mediator variables

|  | **Baseline** | | | | **Four weeks** | | | | **Six months** | | | |  |
| --- | --- | --- | --- | --- | --- | --- | --- | --- | --- | --- | --- | --- | --- |
|  | **Intervention** | | **Control** | | **Intervention** | | **Control** | | **Intervention** | | **Control** | |  |
| **VARIABLES** | **n** | **Mean (SD)** | **n** | **Mean (SD)** | **n** | **Mean (SD)** | **n** | **Mean (SD)** | **n** | **Mean (SD)** | **n** | **Mean (SD)** |  |
|  |  |  |  |  |  |  |  |  |  |  |  |  |  |
| PA self-efficacy  (1-5) | 439 | 2·91 (0·97) | 376 | 2·92 (0·94) | 344 | 2·83 (0·89) | 319 | 2·80 (0·92) |  |  |  |  |  |
| Intentions (1-7) | 435 | 5·38 (1·68) | 375 | 5·37 (1·75) | 343 | 5·42 (1·58) | 321 | 5·09 (1·77) |  |  |  |  |  |
| Outcome expectations (1-5) | 418 | 3·37 (0·62) | 354 | 3·36 (0·64) | 318 | 3·25 (0·67) | 292 | 3·27 (0·60) |  |  |  |  |  |
| Financial motivation  (1-7) | 439 | 1·71 (1·16) | 376 | 1·79 (1·28) | 345 | 2·12 (1·36) | 320 | 2·06 (1·42) |  |  |  |  |  |
| Planning (1-4) | 414 | 2·37 (0·69) | 363 | 2·45 (0·69) | 344 | 2·29 (0·70) | 319 | 2·32 (0·75) | 255 | 2·35 (0·74) | 235 | 2·32 (0·71) |  |
| Social norms (1-7) | 414 | 3·87 (1·20) | 357 | 4·04 (1·14) | 346 | 3·93 (1·16) | 317 | 3·78 (1·29) | 253 | 3·90 (1·13) | 235 | 3·90 (1·12) |  |
| Identified regulation (1-5) | 438 | 3·81 (0·87) | 375 | 3·92 (0·83) | 346 | 3·99 (0·78) | 319 | 3·89 (0·83) | 262 | 3·93 (0·82) | 239 | 3·91 (0·87) |  |
| Integrated regulation (1-5) | 439 | 3·12 (1·13) | 373 | 3·27 (1·11) | 344 | 3·37 (1·08) | 319 | 3·24 (1·10) | 258 | 3·41 (1·10) | 238 | 3·31 (1·12) |  |
| Intrinsic motivation (1-5) | 438 | 3·52 (0·99) | 376 | 3·63 (0·97) | 346 | 3·70 (0·88) | 320 | 3·58 (0·94) | 259 | 3·70 (0·91) | 239 | 3·63 (0·97) |  |
| Habit (1-5) | 437 | 2·89 (1·32) | 375 | 3·08 (1·24) |  |  |  |  | 256 | 3·18 (1·40) | 235 | 2·87 (1·45) |  |
| Workplace norms (1-5) | 439 | 3·20 (0·82) | 377 | 3·20 (0·85) |  |  |  |  | 260 | 3·19 (0·76) | 237 | 3·14 (0·83) |  |
| Recovery self-efficacy (1-4) | 438 | 2·36 (0·82) | 375 | 2·34 (0·79) |  |  |  |  | 261 | 2·41 (0·73) | 238 | 2·41 (0·70) |  |
| Maintenance self-efficacy (1-4) | 438 | 2·79 (0·86) | 376 | 2·77 (0·89) |  |  |  |  | 262 | 2·69 (0·83) | 237 | 2·69 (0·75) |  |
| Outcome satisfaction (1-5) | 404 | 3·85 (0·68) | 352 | 3·87 (0·65) |  |  |  |  | 257 | 3·87 (0·62) | 233 | 3·80 (0·69) |  |
|  |  |  |  |  |  |  |  |  |  |  |  |  |  |

PA: physical activity; SD: standard deviation; ( ) in the variables column indicate the range values for each measure
