# Supplementary material for: Effectiveness and cost-effectiveness of a loyalty scheme for physical activity behaviour change maintenance: results from a cluster randomised controlled trial
Source: Int J Behav Nutr Phys Act. 2018 Dec 12;15:127. doi: 10.1186/s12966-018-0758-1 (PMC6291971; doi:10.1186/s12966-018-0758-1)
Supplement: Supplementary file 10 — Table S5. Results of single mediator models with six month pedometer steps/day as the dependent variable. (DOCX 24 kb) [file 12966_2018_758_MOESM10_ESM.docx]

**Table S5: Results of single mediator models with six month pedometer steps/day as the dependent variable**

| **Hypothesised mediators** | **n** | **Intervention effect on mediator** | | **Association of mediator with PA** | |
| --- | --- | --- | --- | --- | --- |
|  |  | ***a* (SE)** | **P-value** | ***b* (SE)** | **P-value** |
| ***Mediators of initiation^1^*** | | | | | |
| PA self-efficacy | 417 | 0·11 (0·08) | 0·15 | 293 (161) | 0·07 |
| Intentions | 415 | 0·39 (0·18) | **0·03** | -29 (58) | 0·62 |
| Outcome expectations | 363 | -0·04 (0·05) | 0·38 | -44 (143) | 0·76 |
| Financial motivation | 420 | 0·20 (0·15) | 0·19 | 13 (75) | 0·87 |
| Planning | 403 | 0·06 (0·06) | 0·34 | -51 (287) | 0·86 |
| Social norms | 405 | 0·24 (0·09) | **<0·01** | -137 (87) | 0·12 |
| Identified regulation | 417 | 0·14 (0·06) | **0·03** | 94 (282) | 0·74 |
| Integrated regulation | 416 | 0·22 (0·07) | **<0·01** | 106 (231) | 0·65 |
| Intrinsic motivation | 418 | 0·16 (0·06) | **<0·01** | 37 (252) | 0·88 |
| ***Mediators of maintenance^2^*** | | | | | |
| Planning | 382 | 0·09 (0·06) | 0·15 | 547 (180) | **<0·01** |
| Social norms | 382 | 0·08 (0·09) | 0·37 | 262 (94) | **<0·01** |
| Identified regulation | 403 | 0·06 (0·06) | 0·30 | 550 (211) | **<0·01** |
| Integrated regulation | 399 | 0·17 (0·09) | 0·07 | 571 (188) | **<0·01** |
| Intrinsic motivation | 400 | 0·13 (0·07) | 0·06 | 456 (176) | **0·01** |
| Habit | 394 | 0·41 (0·14) | **<0·01** | 482 (105) | **<0·01** |
| Workplace norms | 400 | 0·10 (0·06) | 0·11 | -362 (158) | **0·02** |
| Recovery self-efficacy | 402 | -0·02 (0·07) | 0·83 | 163 (150) | 0·28 |
| Maintenance self-efficacy | 403 | -0·02 (0·08) | 0·84 | 208 (134) | 0·12 |
| Outcome satisfaction | 376 | 0·07 (0·05) | 0·21 | 407 (250) | 0·10 |

^1^Mediators of initiation measured baseline and four weeks; ^2^Mediators of maintenance measured baseline and six months.

NB: results are coefficients and cluster-adjusted standard errors and p-values from single mediator models. Bias-corrected bootstrap confidence intervals for the indirect effect are reported (10,000 reps). IV=Group assignment, MV=follow-up scores of mediators, DV=follow-up scores of outcome (six month pedometer steps/day). All paths are adjusted for strata, season, baseline values of the mediator and baseline pedometer steps/day. Unstandardised coefficients are extracted.

Hypothesised mediators of initiation of PA were collected at baseline and at four weeks post-baseline and included outcome expectations, PA self-efficacy, intention, planning, financial motivation, self-determined motivation (i.e. identified regulation, integrated regulation and intrinsic motivation), and social and workplace norms. Hypothesised mediators of maintenance of PA were collected at baseline and six months and included planning, self-determined motivation, habit, recovery and maintenance self-efficacy, outcome satisfaction, and social and workplace norm
