# Supplementary material for: Effectiveness and cost-effectiveness of a loyalty scheme for physical activity behaviour change maintenance: results from a cluster randomised controlled trial
Source: Int J Behav Nutr Phys Act. 2018 Dec 12;15:127. doi: 10.1186/s12966-018-0758-1 (PMC6291971; doi:10.1186/s12966-018-0758-1)
Supplement: Supplementary file 11 — Table S6. Intervention costs. (DOCX 21 kb) [file 12966_2018_758_MOESM11_ESM.docx]

#### Table S6: Intervention costs

| **Cost Items** | **Unit cost** | **Quantity** | **Total cost for six months** |
| --- | --- | --- | --- |
| **Retail vouchers (Club Marketing Ltd)** | / | / | £21,000 |
| **Equipment** |  |  |  |
| Keyrings | £0·35 | 503 ( 457 participants *(1+10%))^a^ | £117·37^b^ |
| Numbering of key rings | £0·04 | 503 ( 457 participants *(1+10%))^a^ | £16·91 |
| Sensor cost | £151·55 | 70 | £2,792·16^c^ |
| **Staff time and travel** |  |  |  |
| Sensor maintenance (electrical engineer) | £13·33/hour | 26 hours ( 1 hr/week * 26 weeks) | £346·58 |
| Sensor maintenance (travel) | £0·40/mile | 338 miles (13 miles/week *26 weeks) | £135·20 |
| Website maintenance (computer scientist) | £13·33/hour | 78 hours ( 3 hr/week * 26 weeks) | £1,039·74 |
| **Total (six month)** |  |  | **£25,447·96** |
| **Cost per participant in the Intervention Group (n=457)** |  |  | **£55·68** |

Note: a. Only for the participants in the Intervention Group and account for a 10% lost/broken/ replacements.

b. Nine months life span was estimated for key rings, and therefor cost for six months was calculated as a proportion of the nine months total cost.

c. Two years life span for sensor was estimated for the sensors. The cost was annuitized at 3.5% discount rate to estimate the cost of one-year use of the sensors. Formular: E=K/[(1-(1+r)-n)/r]. K=£151.55*70=10,608.50, r=3.5%, n=2. E=5584.32. To estimate the six months cost, half of the first year cost was taken due to no discount within each year.
