# Supplementary material for: Effectiveness and cost-effectiveness of a loyalty scheme for physical activity behaviour change maintenance: results from a cluster randomised controlled trial
Source: Int J Behav Nutr Phys Act. 2018 Dec 12;15:127. doi: 10.1186/s12966-018-0758-1 (PMC6291971; doi:10.1186/s12966-018-0758-1)
Supplement: Supplementary file 12 — Table S7. NHS and social care resource use per participant over six months (complete case). (DOCX 24 kb) [file 12966_2018_758_MOESM12_ESM.docx]

#### Table S7: NHS and social care resource use per participant over six months (complete case)

| **Resource use**  **(No. of visits)** | **Intervention (N=457)** | | | | | |  | **Control (N=396)** | | | | | |  | **p value** |
| --- | --- | --- | --- | --- | --- | --- | --- | --- | --- | --- | --- | --- | --- | --- | --- |
|  | **N** | **Mean** | **SD** | **Median** | **Min** | **Max** |  | **N** | **Mean** | **SD** | **Median** | **Min** | **Max** |  |  |
| GP | 261 | 1·21 | 1·36 | 1 | 0 | 7 |  | 235 | 1·24 | 1·53 | 1 | 0 | 10 |  | 0·78 |
| Nurse | 251 | 0·71 | 2·08 | 0 | 0 | 20 |  | 231 | 0·54 | 1·25 | 0 | 0 | 10 |  | 0·28 |
| Physiotherapist | 251 | 0·51 | 1·49 | 0 | 0 | 8 |  | 232 | 0·76 | 2·77 | 0 | 0 | 23 |  | 0·20 |
| A&E | 250 | 0·10 | 0·38 | 0 | 0 | 3 |  | 230 | 0·07 | 0·29 | 0 | 0 | 2 |  | 0·48 |
| Outpatient | 252 | 0·43 | 0·99 | 0 | 0 | 8 |  | 230 | 0·57 | 1·28 | 0 | 0 | 8 |  | 0·17 |
| Inpatient | 251 | 0·04 | 0·28 | 0 | 0 | 3 |  | 229 | 0·04 | 0·22 | 0 | 0 | 2 |  | 0·98 |
| Nights in Hospital | 250 | 0·16 | 1·86 | 0 | 0 | 28 |  | 226 | 0·13 | 0·86 | 0 | 0 | 7 |  | 0·86 |

A&E: Accident and Emergency; GP: General Practitioner; NHS: National Health Service; SD: standard deviation
